# Microengineered Conductive Elastomeric Electrodes for Long-Term Electrophysiological Measurements with Consistent Impedance under Stretch

**DOI:** 10.3390/s151026906

**Published:** 2015-10-23

**Authors:** Dinglong Hu, Tin Kei Cheng, Kai Xie, Raymond H. W. Lam

**Affiliations:** Department of Mechanical and Biomedical Engineering, City University of Hong Kong, Tat Chee Avenue, Kowloon, Hong Kong, China; E-Mails: dinglhu2-c@my.cityu.edu.hk (D.H.); tinkeicheng@gmail.com (T.K.C.); kaixie2-c@my.cityu.edu.hk (K.X.)

**Keywords:** biopotential, microstructure, nanoparticle, ECG, PDMS

## Abstract

In this research, we develop a micro-engineered conductive elastomeric electrode for measurements of human bio-potentials with the absence of conductive pastes. Mixing the biocompatible polydimethylsiloxane (PDMS) silicone with other biocompatible conductive nano-particles further provides the material with an electrical conductivity. We apply micro-replica mold casting for the micro-structures, which are arrays of micro-pillars embedded between two bulk conductive-PDMS layers. These micro-structures can reduce the micro-structural deformations along the direction of signal transmission; therefore the corresponding electrical impedance under the physical stretch by the movement of the human body can be maintained. Additionally, we conduct experiments to compare the electrical properties between the bulk conductive-PDMS material and the microengineered electrodes under stretch. We also demonstrate the working performance of these micro-engineered electrodes in the acquisition of the 12-lead electrocardiographs (ECG) of a healthy subject. Together, the presented gel-less microengineered electrodes can provide a more convenient and stable bio-potential measurement platform, making tele-medical care more achievable with reduced technical barriers for instrument installation performed by patients/users themselves.

## 1. Introduction

Conductive materials are often applied as electrodes for measurements of biopotential, which involves the voltage variation generated by muscle contraction/relaxation [1]. Further, multiple biopotential electrodes have been long applied with standard configurations to capture physiological information with clinical significance, e.g., electrocardiogram (ECG), electroencephalogram (EEG) and electrooculogram (EOG) [2,3,4]. In particular, ECG is in the form of a transthoracic (across the thorax or chest) interpretation of the electrical activities between multiple pairs of defined spots on the human body across the heart for diagnosis and monitoring of the heart [5,6,7]. However, many of the existing electrodes are wet electrodes (e.g., Ag/AgCl electrodes), requiring additional gels as an interfacial material to increase the dielectric constant between the electrode and skin surface for better measurement [8,9]. Additionally, conductive gels or ionic liquids [10] can also be applied directly to the skin in minimal amounts [11] to further optimize electrical conductivity. Nevertheless, the additional gel could complicate the measurement setup procedures, and sometimes the conductive gel can even cause skin allergy.

Researchers have been working on development of the ‘dry’ biopotential electrodes (*i.e.*, no additional gels are required [12]). For instance, Dias *et al.*, reported a dry electrode composed of iridium oxide (IrO) [13]. Needle-array microstructures fabricated with conductive materials were applied as the invasive electrodes (*i.e.*, the micro-needles pinned into the epidermis) in order to increase the contact area of measurement. Arrays of carbon nanotubes were also applied as the invasive needle-type electrodes, which penetrated into the outer layers of the skin and the stratum corneum to improve the electrical contact [14]. Nevertheless, infection through the minor wounds caused by the needle penetration was an important concern [15,16]. Recently, thin bulk conductive polymers have been applied in the ‘dry’ biopotential sensing, due to the material flexibility for better physical conformability and contact area with the skin surface [17,18,19]. Leleux *et al.* reported a biopotential electrode using conductive polyethylenedioxythiophene doped with polystyrenesulfonate and demonstrated its great potentials in electrophysiological measurements such as EOG, ECG and EEG [9,10,20]. Joeng *et al.*, reported the implementation of long-term electrophysiological measurements using capacitive epidermal electronics [8]. Bulk conductive polydimethylsiloxane (PDMS) was also applied as flexible electrodes for biopotential acquisition [21]. These conductive polymer/nonmetallic electrodes have shown reasonable impedance and biocompatibility, but there are still some issues such as the electrical properties’ variations under the material deformations caused by body movements.

In this paper, we report the development of dry microengineered conductive elastomeric biopotential electrodes for ECG measurement with higher electrical consistence. We chose the PDMS elastomer as the structural material because it is biocompatible, chemically inert, gas/moisture-permeable, and highly deformable. With these material properties, PDMS has become one of the most widely used materials in many biological or biomedical applications [22,23]. Mixing PDMS with other conductive nanoparticles (graphite, carbon black and acetylene black) can achieve the required electrical conductivity for biopotential measurements [24].

## 2. Design and Fabrication of the Conductive Elastomeric Electrodes

**Figure 1:**
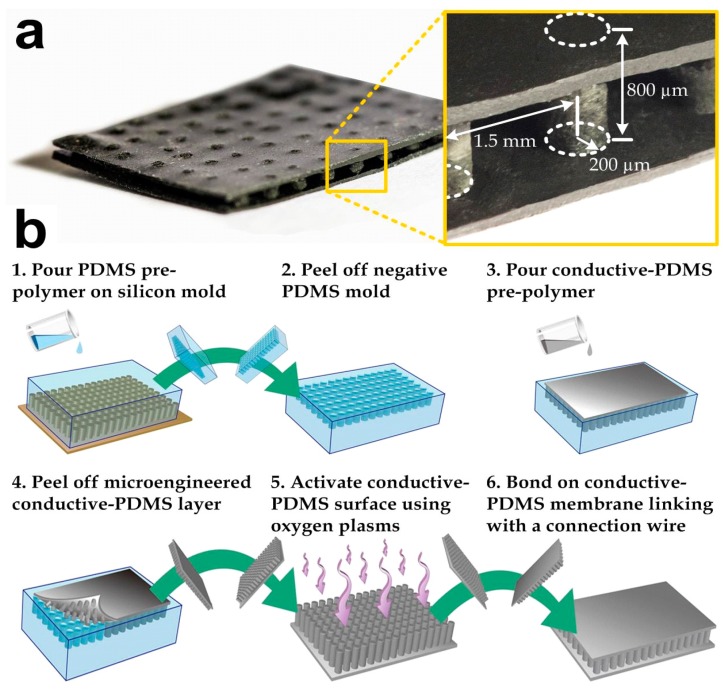
(**a**) Photograph of a microfabricated conductive-polydimethylsiloxane (PDMS) biopotential electrode. A connection wire can be further attached to this electrode for the signal acquisition; (**b**) Key steps in microfabrication of a conductive-PDMS electrode.

As the conductive-PDMS is highly flexible, the physical conformity on human skin can be achieved by the material flexibility, rather than having sharp nano-needles penetrate the skin’s surface. However, body movements of the user can significantly deform the bulk material and subsequently cause variations in its electrical properties. Therefore, we have designed and micro-fabricated the conductive-PDMS material as shown in Figure 1a. The conductive-PDMS microstructures contained an array of non-penetrative micro-pillars (radius: 200 μm; height: 800 μm; and center-center distance: 1.5 mm) sandwiched by two flat membranes (thickness: 100 μm). The conductive-PDMS micro-pillars [25,26] can maintain their shapes and electrical resistances under a lateral stretch of the electrode whereas the material deformation mainly occurs on the upper and lower membranes. We applied a modified micro-replica mold casting process to fabricate the micro-structures as shown in Figure 1b. Our previous investigation has shown that the geometry of micro-pillars can be retained under the stretch of the underlying membrane, where the strain increment propagates only to the lowest portion of each pillar, and therefore the overall material properties can be maintained [25]. Considering that micro-pillars align along the direction of signal transmission, the detected bio-signals from the skin surface/lower membrane, via the micro-pillars and the upper membrane, and to the signal acquisition system become more stable than using the bulk conductive-PDMS as the electrodes.

We fabricated the conductive elastomeric electrode (Figure 1a) by two replica-molding steps of PDMS [27,28,29,30]. Silicon mold masters were first prepared before the PDMS casting. Two silicon wafers were spin-coated with a photoresist (SU-8 100, Microchem, Westborough, MA, USA) with a target height of 100 μm. The photoresist layers were patterned using photolithography and developed to form square bosses. One of the silicon wafers was spin-coated again for an 800-μm photoresist (SU-8 100, Microchem) layer and the patterned SU-8 microstructures were micro-pillars with the same dimensions as our target conductive-PDMS pillars. The mold surfaces were silanized by first activating the surface using an oxygen plasma treatment (Energy: 10 kJ; Harrick plasma cleaner PDC-002, Ithaca, NY, USA) and a molecular layer of trichloro (1H, 1H, 2H, 2H-perfluoro-octyl) silane (Sigma-Aldrich, St. Louis, MO, USA) was deposited, in order to facilitate release of the PDMS from the silicon master in the following molding process. Figure 1b indicates the fabrication process of the conductive elastomeric electrode. For the first round of replica molding process, a PDMS pre-polymer (Sylgard-184, Dow Corning, Auburn, MI, USA) was first prepared by mixing the monomer with the curing agent (with a *w/w* ratio of 10:1). The mixture was stirred for three to five minutes, degassed and poured onto the silicon master (with microstructures) and baked at 80 °C overnight for thorough cure (Step 1). After cutting and peeling the PDMS substrate off from the silicon master (Step 2), this PDMS negative-mold was silanized overnight with trichloro (1H, 1H, 2H, 2H-perfluoro-octyl) silane. The PDMS pre-polymer was prepared again by mixing the monomer and curing agent with the *w/w* ratio of 10:1, and then mixed with selected conductive nanoparticles using a defined *w/w* ratio. The conductive-PDMS mixture was then poured onto the pre-fabricated negative pure-PDMS mold (Step 3). In order to remove the gaps and air trapped in the conductive material substrate, the conductive-PDMS and the mold were degassed and compacted using two PMMA boards (Step 4). The substrate was then placed into an oven and baked at 80 °C overnight for thorough curing. Meanwhile, a flat conductive-PDMS layer was prepared with the silicon master (only with boss) using mixed PDMS with the same nanoparticles. Before baking this flat substrate overnight, an electrical wire was inserted into the conductive-PDMS pre-polymer for the signal transmission purpose. To finish the fabrication, both the flat and micro-pillar conductive-PDMS substrates were peeled off and chopped. Oxygen plasma treatment was applied on their surfaces to covalently bond them together (Steps 5–6).

## 3. Electrical Conductivity for Different Concentrations of the Selected Nanoparticles in PDMS

**Figure 2:**
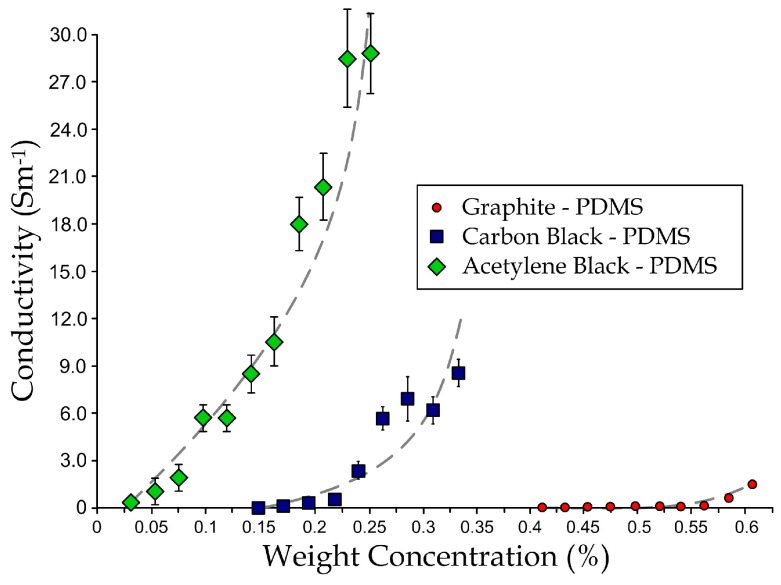
Relationships between concentration and conductivity for graphite-PDMS, carbon black-PDMS, and acetylene black-PDMS.

In order to optimize the composite candidates as bio-potential electrodes, we performed a series of experiments on conductive-PDMS specimens with the same size and shape but different nanoparticle types and concentrations. We considered three nanoparticle candidates: acetylene black, carbon black, and graphite. We measured the electrical resistances of these specimens by clamping the two ends of each sample using crocodile clips connecting to an LCR meter (VICTOR VC9808+). The electrical conductivity *C* of each specimen was calculated by *C* = *L*/(*RA*), where *R* is measured sample resistance, *A* is cross-sectional area and *L* is sample length. Figure 2 shows that the conductivity increases with the weight concentrations of the nanoparticles. However, it should be mentioned that an excessively high concentration of the nanoparticles simultaneously reduces the material strength and durability maintained by the cross-linkage level of PDMS molecules, therefore the feasible concentration of nanoparticles has an upper limit. Among the three nanoparticle candidates, it can be observed that acetylene black is the most effective supplement to enhance the composite conductivity, while graphite has the lowest effectiveness. For instance, acetylene black-PDMS can achieve a conductivity of ~30 Sm^−1^ with a weight concentration of ~24%, whereas graphite-PDMS can induce a conductivity of only ~2 Sm^−1^ with a high weight concentration of ~60%. Thus, we have chosen 20% (*w/w*) of acetylene black in PDMS as the target substrate material for the microengineered electrode development. Our results indicate that acetylene black can induce higher conductivity to the PDMS substrate. In the electrode fabrication, we chose the weight ratio of acetylene black in PDMS to be 20% in order to retain sufficient material strength. Importantly, there are also medical grade versions of the selected materials available, e.g., *Silastic* biomedical grade ETR elastomers (Q7 series, Dow Corning, Auburn, MI, USA) [31] for silicone and Unipore Black LC902 (Adina Cosmetic Ingredients, Kent, UK) for acetylene black [32]. Further development of the conductive silicone using these medical grade materials, as replacements will guarantee long-term biocompability of the microengineered biopotential electrodes in clinical applications.

## 4. Simulation of Resistance of the Microengineered Electrode under Different Stretch Levels

**Figure 3:**
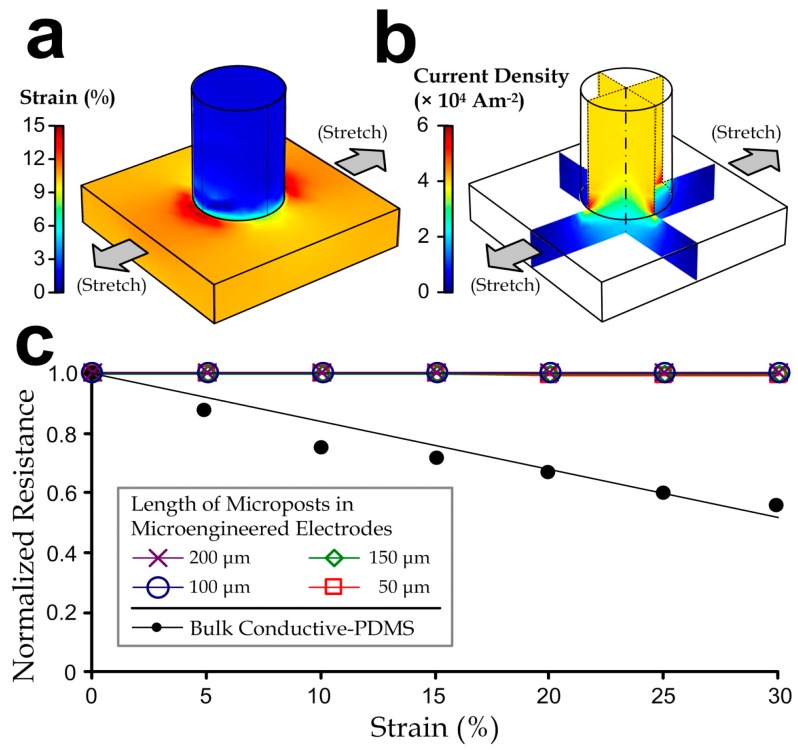
(**a**) Strain profiles; (**b**) Current density profiles of a representative microengineered conductive-PDMS electrode with a micro-pillar half-height of 400 μm at a 10% uniaxial stretch; (**c**) Normalized resistance of bulk conductive-PDMS and microengineered electrodes with different micro-pillar heights as functions of strain.

As aforementioned, disturbances such as body movements and compression by external objects can cause deformation of the flexible electrodes, changes in the electrode conductivity, and additional signal variations. To analyze this effect, we have performed simulations for the bulk conductive-PDMS and microengineered electrodes with a range of micro-pillar heights to obtain the corresponding strain and current profiles as a function of the tensile strain (Figure 3a). We used commercial simulation software (COMSOL 4.3b) in order to analyze the physical properties and electricity profiles for different dimensional parameters of the electrode design. Intrinsic material properties of the acetylene black-PDMS (e.g., elastic modulus: 17 MPa; density: 0.97 kg/m^3^, Poisson’s ratio: 0.45; relative permittivity: ~2.8, and electrical conductivity: 23.7 Sm^−1^) were determined by our tests on the bulk material (~20% weight ratio of acetylene black in PDMS). We applied the Neo-Hookean hyper-elastic model to describe the material deformation because the composite material contained mainly the elastomeric PDMS. We considered the lower half of one micro-pillar region as our simulation model, due to the repeating micro-pillar structures in the electrode, and the symmetry between the upper and lower halves of one micro-pillar region. The model is a cylinder (radius: 200 µm; half-height: 0–400 µm) fixed on top of a square block (side length: 1.5 mm; height: 100 µm). On the other hand, we also simulated the resistance of a bulk conductive-PDMS with dimensions equivalent to a pillar region with a pillar height 400 µm, *i.e.*, 1.5 mm (w) × 1.5 mm (l) × 500 µm (h). We then imposed a range of defined lateral strains (0%–30%) on region boundaries of both the micro-pillar model and the bulk PDMS model, and further examined for their strain profiles and the electrical resistance along the “height” direction of the models. We set the voltage difference between the top and bottom surfaces of the model to be 1 V and computed for the current density (unit: A/m^2^; Figure 3b) and the electrical resistance accordingly. Notably, the resistance of a single pillar region on the electrode should be double that of the simulation values.

We looked for changes of the overall electrode resistance as functions of lateral strain levels (0%–30%), which were normalized as the ratio to the unstretched status. Figure 3c shows that the “normalized” resistance of the microengineered electrodes (the ratio relative to the resistance of the unstretched material) is consistent under the mechanical stretch, whereas the bulk conductive-PDMS has a relative resistance reduction rate of ~0.02 per every 1% of strain. Therefore, biopotential signals measured by the microengineered electrode should be more consistent upon stretches caused by body movements in practical electrophysiological acquisition implementations.

## 5. Characterization for the Bulk and Microengineered Electrodes

**Figure 4:**
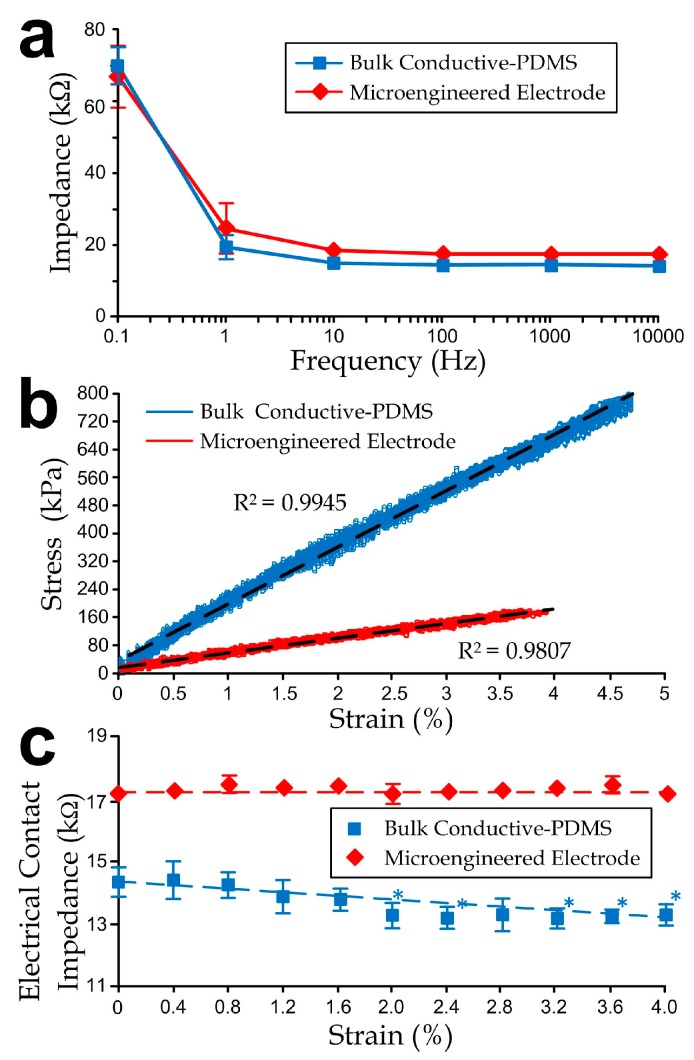
(**a**) Impedance curves of bulk and microengineered electrodes as functions of signal frequencies; (**b**) Stress-strain curves and (**c**) Electrode contact impedance of the electrodes as functions of tensile strains. The asterisks indicate significant changes (*p*-value < 0.05) from the unstretched case, *i.e.*, the impedance at strain = 0%, using the Student’s *t*-test. All error bars in sub-figures represent standard deviations.

We have performed measurements to characterize both the bulk and microengineered conductive PDMS electrodes (1 cm (W) × 1 cm (L) × 1 mm (H)). We selected the composite of 20% acetylene black in PDMS as the substrates for the bulk and microengineered materials. The electrode contact impedances (considered as the reference impedance at a 20 Hz, as the signals below this frequency already cover the major waveforms [33,34,35]) of both electrode types were sufficiently low (bulk: 14 kΩ; microengineered: 17.5 kΩ as shown in Figure 4a) to fulfill requirements of electrophysiological measurement applications (<20 kΩ for a root-mean-square signal noise of <5 µV at 30 Hz bandwidth [35]).

Further, we investigated the contact impedance variations under different uniaxial tensile strain levels of the conductive-PDMS electrodes. We applied stretches on the conductive-PDMS specimens (length: 5 cm; width: 1 cm; thickness: 1 mm) using an electromechanical material testing system (RT/30; MTS Systems Corporation, Eden Prairie, MN, USA) at a rate of 1 mm/min for a total distance of 1 mm (5% strain), and we simultaneously measured the conductivity along the direction of substrate thickness. Considering that the metal fixtures of the mechanical tester are conductive, we isolated both ends of the mounted samples by polymeric sealing films (cat# A5596, Sigma-Aldrich). The electrical resistances of the conductive-PDMS at different strains were measured at different distances of the stretching. The corresponding equivalent uniaxial elastic moduli (Figure 4b) of the bulk and microengineered electrodes were 17 MPa and 4.45 MPa respectively, indicating that the microfabricated structures provided physical flexibility and conformity of the skin-electrode contact. Figure 4c indicates that the embedded micropost structures could help with maintaining the impedance consistency by eliminating the strain-induced impedance variation (~0.32 kΩ/%) as observed in the bulk electrode measurement. If we further consider the relative impedance reduction rate, the measured value ~0.22 per 1% of strain agrees with the simulation (~0.022 per 1% of strain). Under stretch, only minor fluctuations on the electrical contact impedance of the bulk conductive-PDMS (within ±5% of the average level), and microengineered electrode (within ±1% of the average level).

## 6. 12-Lead ECG Measurement System Integrated with the Microengineered Electrodes

**Figure 5:**
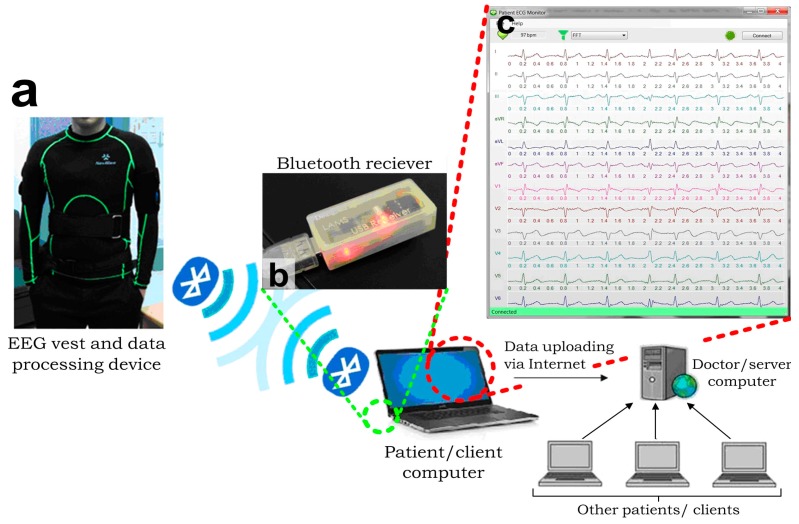
Architecture of the integrated ECG measurement system [36]. (**a**) EEG vest and data processing device detects and transmits the EEG signals; (**b**) A Bluetooth module receives the transmitted ECGs and passes the data to a client computer, in which (**c**) An interface program is developed and installed. This interface program performs ECG recording, digital filtering and transmission via the internet to a server computer, which can handle signals from multiple clients simultaneously.

Additionally, we have applied the microengineered conductive-PDMS dry electrodes to implement the clinically recognized 12-lead ECG acquisition by developing a fully functionally ECG vest (Figure 5a) and the corresponding measurement system. A comprehensive ECG acquisition system was established by integrating the ECG vest with Bluetooth and Internet communication channels for the continuous remote-monitoring of patients’ ECGs as described in Figure 5a. Briefly, the data flow started with the signal acquisition of the ECG vest. The microengineered electrodes embedded on the ECG vest were connected to a portable data acquisition device. This portable device preliminarily processed the ECG signals by hardware signal filtering and transmission to an external computer via Bluetooth. We developed a Bluetooth receiver (Figure 5b) connecting to the external client computer via a USB port using the UART data transmission protocol for the continuous data collection. We also developed a customized graphic user interface (GUI) program (Figure 5c) in the client computer to record the ECG signals and further upload the ECGs to a server computer via the Internet to simultaneously consolidate multiple patients’ records (e.g., name, gender, contact information, ECGs at different dates, and medical records) as a central database. The server computer could be physically located in the hospital such that the doctor can monitor and fetch the ECG records of multiple patients. Each patient with an EEG vest and a client computer installed in his/her home could view his/her own ECG signals locally, while doctors could access multiple patients’ ECG data for further analysis and diagnosis, either in real time or offline through the server computer.

**Figure 6:**
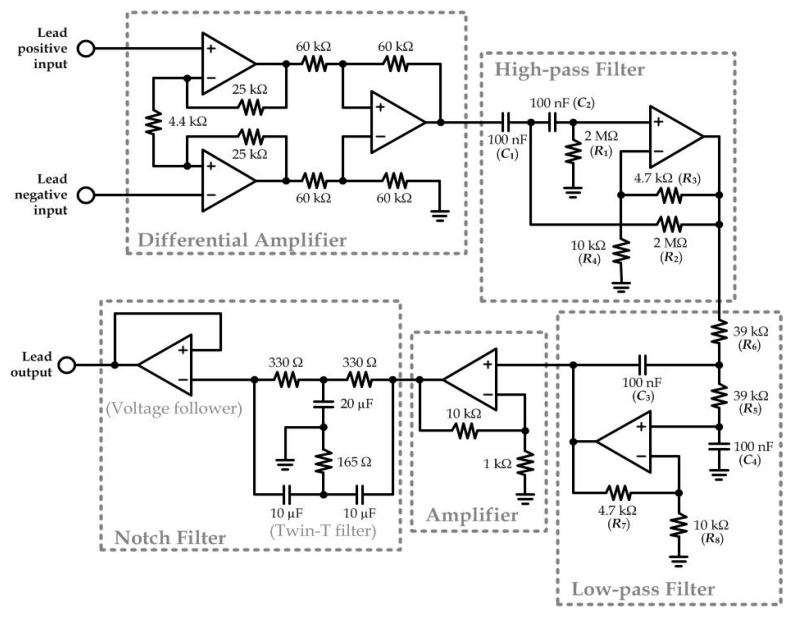
Circuit schematics for detection of each lead of the 12-lead ECG signals.

Detection of biopotential from human skin should involve the potential signals in the range of <1 mV, which requires relatively sensitive electrodes with good conductivity, a high quality signal amplifier, and a signal filter. In the portable ECG acquisition device mentioned previously, we have included a biopotential amplifying and filtering module for signal acquisition of each ECG electrode. For each lead of the measurement, an individual circuitry was integrated from multiple operational amplifiers (UA741CN) as shown in Figure 6. First, a differential amplifier was applied to amplify the potential difference between the two body positions defined by the lead. Two second-order Sallen-Key filters (one high-pass filter and one low-pass filter) were applied to function together as a band-pass filter in order to extract the frequency range representative to the ECG signals (lower cut-off frequency: 0.2 Hz; higher cut-off frequency: 100 Hz). The transfer functions of the high-pass and low-pass filters were described in Equations (1) and (2), respectively:

(1) $$H\left(s\right)\;=\;(1\;+R_{4}/R_{3})\;\times s^{2}/[s^{2}+s(1/R_{2}C_{1}+\;1/R_{2}C_{2})\;+\;1/R_{1}R_{2}C_{1}C_{2}]$$

(2) $$H\left(s\right)\;=\;(1\;+R_{7}/R_{8})\;\times \;(1/R_{5}R_{6}C_{3}C_{4})/[s^{2}+s(1/R_{5}C_{3}+\;1/R_{6}C_{3})\;+\;1/R_{5}R_{6}C_{3}C_{4}]$$

The following amplifier circuit was a simple non-inverting amplifier (Gain: 11) that amplifies the output from the low-pass filter. Next, a twin-T filter and a voltage follower composed as a Notch filter to eliminate any possible noise caused by the 50 Hz alternating current signals from other appliance driven by the building power system.

**Figure 7:**
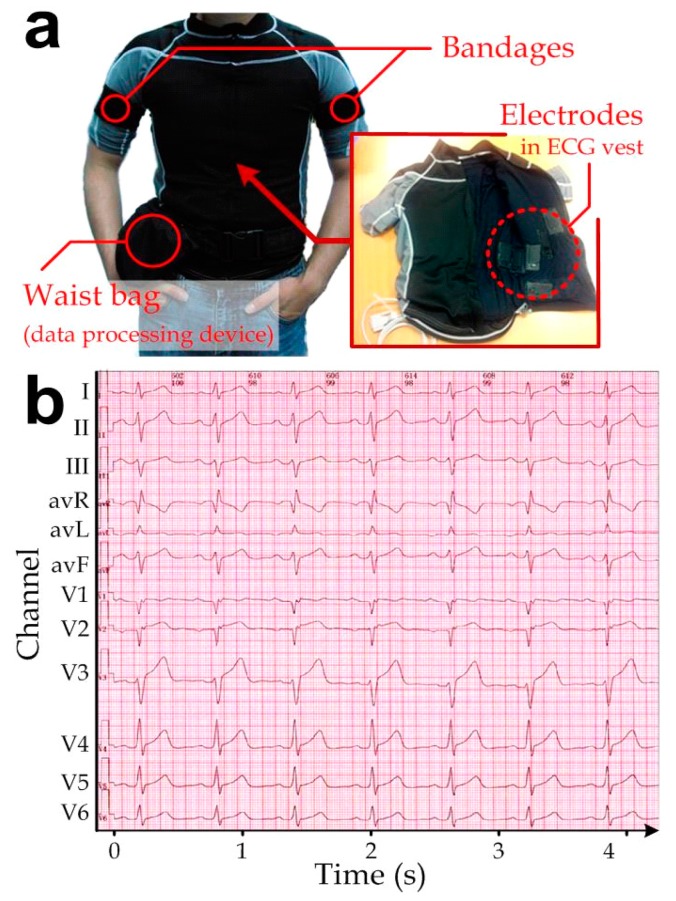
(**a**) ECG vest embedded with the microengineered conductive-PDMS electrodes (inset) on the inner surface of the clothing. Bandages on the arms were used to fix the electrodes LA and RA in positions. A waist bag contained circuitry for data acquisition, signal processing and wireless transmission; (**b**) Representative 12-lead ECG measured by the ECG vest.

As defined by the 12-lead ECG arrangement, 10 electrodes were embedded onto the inner side of the vest: two on the left and right shoulders (LA and RA), two on the two sides of the waist (LL and RL), and six on the rib cage across the chest (V1–V6). Only bandages were required to tighten the forearms (LA and RA) and waist (LL and RL) for the skin-electrode contact. A zipper was added in the front of the vest so that the wearer could put on the vest more easily. We also added a side waist bag (Figure 7a) for carrying the circuitry for data acquisition, processing, and wireless transmission. The integration of multiple electrodes as a single-piece vest largely simplified the installation procedures. In our implementation, we measured the ECG while the subject (a healthy 24-year-old man) was walking (*i.e.*, with waist muscular movements and physical vibration generated). The ECG measured with the microengineered electrodes (Figure 7b) should be equivalent with the existing ECG measurement systems applied in hospitals, certified by a medical cardiologist (as mentioned in the acknowledgements section). Furthermore, qualified ECG can be captured by the integrated measurement system over the 1-year implementation because of the minor variation in the electrical contact impedance of the electrodes (0.64%). Therefore, the microengineered ECG electrodes can be applied for the long-term electrophysiological measurements.

**Figure 8:**
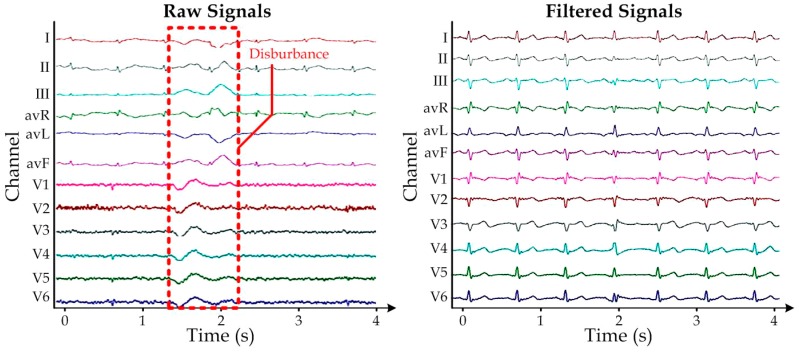
Representative (**a**) Raw 12-lead ECG measurement with disturbance due to body movements; (**b**) The corresponding filtered signals, which have been scaled for better visualization.

To further improve the captured signal quality, we implemented additional digital time-invariant band-pass linear filtering on each lead-signal of the measured ECG, by including the corresponding resolved difference equation in the GUI. In this stage, we chose the lower and higher cut-off frequencies to be 1 Hz and 100 Hz, respectively. Figure 8 demonstrates that the filtering algorithm can effectively remove the unwanted disturbance caused by the body movements in the measured raw 12-lead ECG signals (left sub-figure). A representative filtered ECG is shown in the right sub-figure of Figure 8. Together, this reported highly portable ECG measurement system should provide further potential applications as healthcare and health monitoring products in dynamic locations (e.g., hospitals, first-aid stations, and ambulances).

## 7. ECG Measurement during Human Motions

**Figure 9:**
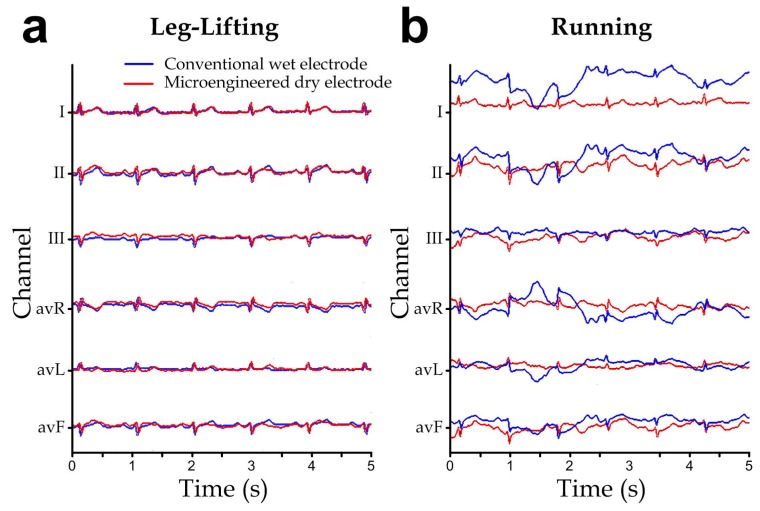
ECG signals simultaneously measured with the conventional wet electrodes (*blue*) and the microengineered electrodes (*red*) during different human motions: (**a**) Walking. (**b**) Running.

Furthermore, we have simultaneously measured the ECGs using both the conventional wet electrodes and the microengineered dry electrodes connected to our ECG acquisition system, in order to compare the measurement performance between the two electrode types. The wet electrodes and microengineered electrodes were placed next to each other to measure the same subject’s ECG. Considering that our ECG acquisition system had only limited number of input channels, 6-lead ECG (channels: I, II, III, avR, avL and avF) [37] were adopted instead of the full 12-lead ECG. We captured the subject’s ECG during his body movements of (1) leg-lifting (*i.e.*, the subject lifted his left and right legs alternatively) and (2) running, and the representative ECGs are shown in Figure 9a,b, respectively. Both the electrode types showed reasonable and comparable signal waveforms under the limb motion during the leg-lifting experiment. Notably, we placed all the electrodes in the torso region and therefore the signal acquisition was less sensitive to muscular activities of the legs. On the other hand, the ECGs during running demonstrated signal artifacts caused by the more rigorous torso muscular movements, which cannot be removed fully by the signal filtering circuit, for both electrode types. Yet the microengineered dry electrodes showed the relatively less signal disturbance, probably because microengineered electrodes could maintain more consistent electrical properties upon deformation caused by the subject’s body movements. Although it is well known that motion artifact is an unavoidable signal distortion in ubiquitous ECG recording, minimizing the signal disturbance by applying the microengineered electrodes should facilitate the downstream ECG signal extraction by the related adaptive filtering algorithms that are developed based on techniques such as time averaging, wavelet transform [38], least-mean-squares, recursive least-squares [39], and fuzzy-rules [40].

## 8. Conclusions

In this paper, we report a microengineered conductive-PDMS electrode for human electrophysiological measurement without the addition of gels. To reduce the changes in the electrical conductivity caused by mechanical stretching of the electrode during body movements of the user, we designed and configured the conductive-PDMS as an array of micro-pillars embedded between two layers. The electrode has been fabricated by micro-replica molding of conductive-PDMS. Based on our characterization of the composites of different nanoparticles (acetylene black, graphite, and carbon black) in PDMS, we chose acetylene black with a 20% weight ratio in PDMS as the substrate material for the sufficiently low skin contact impedance (17.5 kΩ) and rigidity (4.45 MPa). Altogether, the reported microengineered conductive-PDMS electrodes have a great potential for extended biomedical applications such as the long-term electrophysiological diagnosis (e.g., electroencephalography (EEG), and electrooculography (EOG)) and remote medical care or healthcare of general users at home, due to the measurement consistency and simple electrode placement scheme resultant from embedding the electrodes into clothing and other gadgets.
